# A Damping Grid Strapdown Inertial Navigation System Based on a Kalman Filter for Ships in Polar Regions

**DOI:** 10.3390/s17071551

**Published:** 2017-07-03

**Authors:** Weiquan Huang, Tao Fang, Li Luo, Lin Zhao, Fengzhu Che

**Affiliations:** College of Automation, Harbin Engineering University, Harbin 150001, China; huangweiquan@hrbeu.edu.cn (W.H.); luoli1022@hrbeu.edu.cn (L.L.); zhaolin@hrbeu.edu.cn (L.Z.); 13339306757@163.com (F.C.)

**Keywords:** grid SINS, Schuler periodic oscillation, Kalman filter, level damping

## Abstract

The grid strapdown inertial navigation system (SINS) used in polar navigation also includes three kinds of periodic oscillation errors as common SINS are based on a geographic coordinate system. Aiming ships which have the external information to conduct a system reset regularly, suppressing the Schuler periodic oscillation is an effective way to enhance navigation accuracy. The Kalman filter based on the grid SINS error model which applies to the ship is established in this paper. The errors of grid-level attitude angles can be accurately estimated when the external velocity contains constant error, and then correcting the errors of the grid-level attitude angles through feedback correction can effectively dampen the Schuler periodic oscillation. The simulation results show that with the aid of external reference velocity, the proposed external level damping algorithm based on the Kalman filter can suppress the Schuler periodic oscillation effectively. Compared with the traditional external level damping algorithm based on the damping network, the algorithm proposed in this paper can reduce the overshoot errors when the state of grid SINS is switched from the non-damping state to the damping state, and this effectively improves the navigation accuracy of the system.

## 1. Introduction

Polar navigation technology is the basic condition for ships sailing in polar region safely and reliably [1,2]. Inertial navigation system has been the first choice for ships sailing in polar regions as a result of being autonomous, continuous, and comprehensive [3,4,5,6,7]. The working principle of the SINS is that the gyroscopes and accelerometers are strapped on the carrier. The gyroscopes and accelerometers measure the angular motion and linear motion of the carrier, respectively, then the navigation computer calculates the position, velocity, and the attitude of the carrier through the outputs of the gyroscopes and accelerometers. The lines of longitude tend to converge to a point in high latitudes, and the fictitious graticules change from rectangular in low latitudes to triangular in high latitudes, and because of this, the common SINS based on the geographic coordinate system cannot work properly [8,9,10]. Although the wander azimuth inertial navigation system and the free azimuth inertial navigation system can finish the calculation of the attitude and position direction cosine matrix [11,12], there are singular values when extracting the wander azimuth angle and longitude from the position direction cosine matrix [13]. Aiming at the shortcomings of the three kinds of mechanizations mentioned above, transversal SINS based on a transversal coordinate system and grid SINS based on a grid coordinate system are two main research hotspots to solve the problems of ships sailing in polar regions. The transversal SINS mechanization is built with the hypothesis that the earth is a standard sphere, obviously, this assumption brings the principle error to the design of transversal SINS mechanization [14]. However, the principle error doesn’t exist in grid SINS mechanization, so the grid SINS mechanization can be the ideal mechanization for ships working in polar regions.

Due to the presence of measurement errors of the inertial measurement units (IMU) and other error sources, the grid SINS mechanization drifts with time and includes three kinds of periodic oscillation errors, the so-called 84.4 min Schuler, 24 h earth, and Foucault periodic oscillation [15,16,17,18,19]. These periodic oscillation errors are detrimental to the high-precision navigation system over a long time [20,21,22,23], so it is necessary to adopt some methods to suppress these kinds of errors, such as damping technology. Aiming ships which have the external reference information to conduct a system reset regularly in a certain period of time, suppressing the Schuler periodic oscillation is an effective way to enhance navigation accuracy, the so-called level damping technology. Traditional level damping technology includes internal level damping technology and external level damping technology. In the absence of external reference velocity, the internal level damping technology can suppress the Schuler oscillation errors, however, the internal level damping technology is sensitive to the maneuvering of the ships [24,25]. In order to overcome the shortcomings of the internal level damping technology, when the external device such as the Doppler Velocity Log (DVL) can provide an accurate reference velocity, the external level damping technology can suppress the Schuler oscillation errors with the aid of the reference velocity, and this method is not sensitive to the maneuvering of the ships [26]. Traditional external level damping technology suppresses the Schuler periodic oscillation by adding the network to the level circuit of the system. Like the common SINS, when the work state of the grid SINS is switched from the non-damping state to the damping state, big overshoot errors are introduced to the system [27]. The overshoot phenomenon is extremely unfavorable to the improvement of navigation accuracy, and some methods must be adopted to solve this problem.

Aiming at the shortcomings of the traditional external level damping algorithm, a new external level damping algorithm based on a Kalman filter is proposed in this paper. Connecting with the practical situation, first, the criterion to judge if the velocity measured by DVL is available is built, and when the external velocity is judged to be available, the error model of the external velocity can be simplified as a constant in a certain period of time; second, the Kalman filter, based on a grid SINS error model, is established. When the external velocity error contains constant error, only grid level attitude error angles can be accurately estimated, and then the estimated level attitude error angles are used to calibrate the system. Compared with the traditional external level damping algorithm, the proposed algorithm can not only suppress the Schuler periodic oscillation, but also restrain the overshoot errors when the work state of grid SINS is switched from the non-damping state to the damping state. In the following sections of the paper, the new algorithm proposed in this paper will be called KF-DGSINS, and the traditional external level damping algorithm will be called T-DGSINS. KF represents Kalman filter; T represents traditional; D represents damping; GSINS represents grid strapdown inertial navigation system.

## 2. Grid SINS Mechanization

This section first introduces some common coordinate systems and the transformation relationships between each two coordinate systems, and then the grid SINS mechanization is given.

### 2.1. The Definitions of Common Coordinate Systems

The frequently-used coordinated systems in this paper are the geocentric inertial coordinate system i, earth centered earth fixed (ECEF) coordinate system e, body coordinate system b, and geographic coordinate system T. The detailed definitions of these coordinate systems can be seen in reference [24].

**Figure 1:**
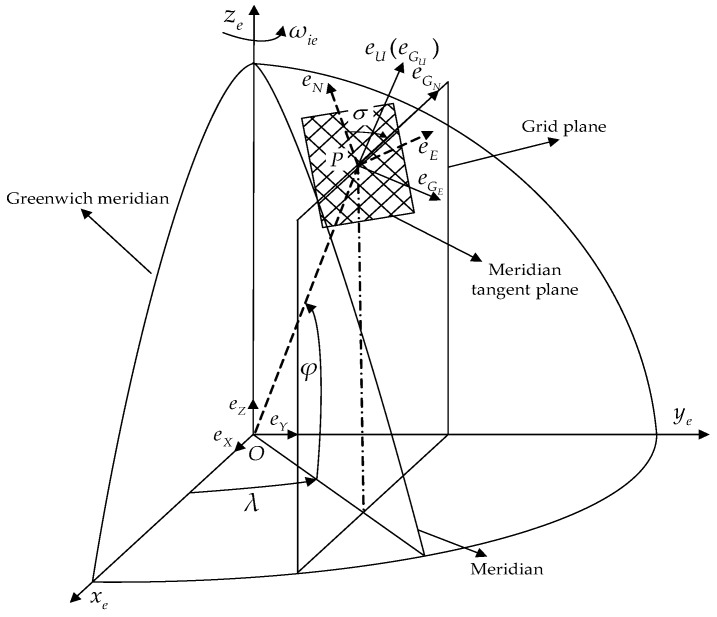
Grid coordinate system G.

The navigation coordinate system of the grid SINS is grid coordinate system G. As shown in Figure 1, when the ship moves to the point P, the grid coordinate system is defined as follows: Grid plane: the parallel plane of the Greenwich plane which passes through the point P; Meridian tangent plane: local horizontal plane which passes through the point P. The grid north axis $G_{N}$ is the intersecting line of the grid plane and meridian tangent plane. The grid up axis $G_{U}$ coincides with the up axis of the geographic coordinate system which passes through the point P. Grid east axis $G_{E}$ lies in the local horizontal plane and is perpendicular to the grid north axis. $G_{E}$, $G_{N}$, and $G_{U}$ constitute the right-handed coordinate system. Obviously, the grid coordinate system is a local horizontal coordinate system, and $G_{N}$ is used as the reference of the grid course angle.

### 2.2. The Transformation Relationships of Common Coordinate Systems

As shown in Figure 1, the angle between $G_{N}$ and the geographic north is called the grid azimuth angle σ. So, the transformation matrix from T frame to G frame is described as:

(1) $$C_{T}^{G}=\left[\begin{array}{ccc} \cos\sigma & -\sin\sigma & 0\\ \sin\sigma & \cos\sigma & 0\\ 0 & 0 & 1 \end{array}\right]$$

φ and λ represent the latitude and longitude of the point P. The sine and cosine of the grid azimuth angle σ can be described as:

(2) $$\sin\sigma =\frac{\sin\varphi \sin\lambda }{\sqrt{1-\cos^{2}\varphi \sin^{2}\lambda }}$$

(3) $$\cos\sigma =\frac{\cos\lambda }{\sqrt{1-\cos^{2}\varphi \sin^{2}\lambda }}$$

The transformation matrix from e frame to T frame is described as:

(4) $$C_{e}^{T}=\left[\begin{array}{ccc} -\sin\lambda & \cos\lambda & 0\\ -\sin\varphi \cos\lambda & -\sin\varphi \sin\lambda & \cos\varphi \\ \cos\varphi \cos\lambda & \cos\varphi \sin\lambda & \sin\varphi \end{array}\right]$$

The transformation matrix from e frame to G frame is described as:

(5) $$\begin{array}{l} C_{e}^{G}=C_{T}^{G}C_{e}^{T}=\left[\begin{array}{ccc} \cos\sigma & -\sin\sigma & 0\\ \sin\sigma & \cos\sigma & 0\\ 0 & 0 & 1 \end{array}\right]\left[\begin{array}{ccc} -\sin\lambda & \cos\lambda & 0\\ -\sin\varphi \cos\lambda & -\sin\varphi \sin\lambda & \cos\varphi \\ \cos\varphi \cos\lambda & \cos\varphi \sin\lambda & \sin\varphi \end{array}\right]\\ =\left[\begin{array}{ccc} -\cos\sigma \sin\lambda +\sin\sigma \sin\varphi \cos\lambda & \cos\sigma \cos\lambda +\sin\sigma \sin\varphi \sin\lambda & -\sin\sigma \cos\varphi \\ -\sin\sigma \sin\lambda -\cos\sigma \sin\varphi \cos\lambda & \sin\sigma \cos\lambda -\cos\sigma \sin\varphi \sin\lambda & \cos\sigma \cos\varphi \\ \cos\varphi \cos\lambda & \cos\varphi \sin\lambda & \sin\varphi \end{array}\right] \end{array}$$

### 2.3. Grid SINS Mechanization

The navigation frame is the grid frame, and the differential equation of strapdown attitude matrix for grid SINS is:

(6) $${\dot{C}}_{b}^{G}=C_{b}^{G}\Omega_{Gb}^{b}$$

where $\Omega_{Gb}^{b}$ is a skew-symmetric matrix related to $\omega_{Gb}^{b}$, and $\omega_{Gb}^{b}$ is obtained as below:

(7) $$\omega_{Gb}^{b}=\omega_{ib}^{b}-\omega_{iG}^{b}=\omega_{ib}^{b}-C_{G}^{b}(\omega_{ie}^{G}+\omega_{eG}^{G})$$

$\omega_{ie}^{G}$ is described as below:

(8) $$\omega_{ie}^{G}=C_{T}^{G}\omega_{ie}^{T}=\left[\begin{array}{ccc} \cos\sigma & -\sin\sigma & 0\\ \sin\sigma & \cos\sigma & 0\\ 0 & 0 & 1 \end{array}\right]\left[\begin{array}{c} 0\\ \omega_{ie}\cos\varphi \\ \omega_{ie}\sin\varphi \end{array}\right]=\left[\begin{array}{c} -\omega_{ie}\cos\varphi \sin\sigma \\ \omega_{ie}\cos\varphi \cos\sigma \\ \omega_{ie}\sin\varphi \end{array}\right]$$

where $\omega_{ie}$ is the rotational angular velocity of the earth. $\omega_{eG}^{G}$ is described as $\omega_{eG}^{G}=P_{3\times 2}*{\left[\begin{array}{cc} V_{E}^{G} & V_{N}^{G} \end{array}\right]}^{T}$. $V_{E}^{G}$, $V_{N}^{G}$ are east and north velocity in grid frame, and $P_{3\times 2}$ is obtained as:

(9) $$P_{3\times 2}=\left[\begin{array}{cc} (\frac{1}{R_{Mh}}-\frac{1}{R_{Nh}})\sin\sigma \cos\sigma & -(\frac{\cos^{2}\sigma }{R_{Mh}}+\frac{\sin^{2}\sigma }{R_{Nh}})\\ \frac{\sin^{2}\sigma }{R_{Mh}}+\frac{\cos^{2}\sigma }{R_{Nh}} & -(\frac{1}{R_{Mh}}-\frac{1}{R_{Nh}})\sin\sigma \cos\sigma \\ \frac{\sin\lambda \cos\varphi }{\sqrt{1-\cos^{2}\varphi \sin^{2}\lambda }}(\frac{1}{R_{Mh}}-\frac{1}{R_{Nh}})\sin\sigma \cos\sigma & -\frac{\sin\lambda \cos\varphi }{\sqrt{1-\cos^{2}\varphi \sin^{2}\lambda }}(\frac{\cos^{2}\sigma }{R_{Mh}}+\frac{\sin^{2}\sigma }{R_{Nh}}) \end{array}\right]$$

where $R_{Mh}=R_{M}+h$ and $R_{Nh}=R_{N}+h$. $R_{M}$ and $R_{N}$ are radius of curvature in meridian and prime vertical, and h is the altitude of the ship. For ships, h is set as zero.

The differential equation of velocity for grid SINS is:

(10) $${\dot{V}}^{G}=C_{b}^{G}f^{b}-(2\omega_{ie}^{G}+\omega_{eG}^{G})\times V^{G}+g^{G}$$

where $g^{G}={\left[\begin{array}{ccc} 0 & 0 & -g \end{array}\right]}^{T}$, g represents the constant of the gravitational acceleration. $V^{G}=\left[\begin{array}{ccc} V_{E}^{G} & V_{N}^{G} & 0 \end{array}\right]$ is the ship’s velocity within grid frame.

The position of the ship is described as the ${\left(\begin{array}{ccc} x & y & z \end{array}\right)}^{T}$ in e frame. $R^{e}={\left(\begin{array}{ccc} x & y & z \end{array}\right)}^{T}$, and the differential equation of position for grid SINS is:

(11) $${\dot{R}}^{e}=C_{G}^{e}V^{G}$$

where $C_{G}^{e}$ is the transpose of $C_{e}^{G}$.

## 3. The KF-DGSINS Algorithm

**Figure 2:**
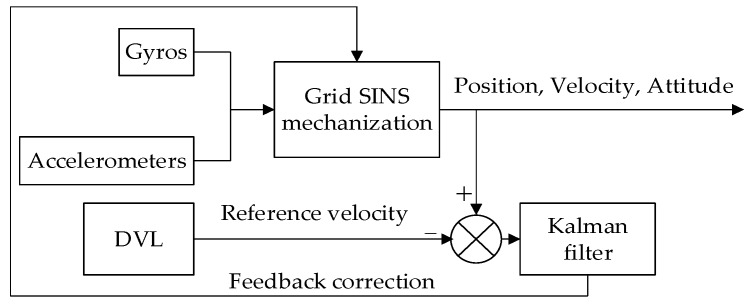
Schematic diagram of the KF-DGSINS.

Based on the optimal estimation theory and feedback correction technology, the schematic diagram of the KF-DGSINS is shown in Figure 2. As shown in Figure 2, gyroscopes and accelerometers measure the angular motion and linear motion of the ship, respectively, and the position, velocity, and the attitude of the ship are calculated through the grid SINS mechanization. The system error states are estimated by Kalman filter with the aid of a reference velocity measured by DVL, and then the Schuler oscillation errors can be effectively suppressed by correcting some error states of the Kalman filter. This section first introduces the error model of the marine grid SINS, then the Kalman filtering and feedback correction technology are described in detail.

### 3.1. The Error Model of the Marine Grid SINS

Establishing an accurate error model is the premise of Kalman filtering. Nonlinear filter algorithms such as EKF, PF, and UKF has been widely studied in the field of integrated navigation system [28], when the real-time requirement is satisfied, the accuracy of the system can be increased. In order to meet the needs of the engineering, the error model is established with the assumptions: the attitude error angle is extremely small; measurement errors of the IMU are simplified as the sum of the constant and the white noise. Based on these two assumptions, the nonlinear factor of the system can be ignored, and the linear error model can be established. Reference [16] has given the airborne polar grid SINS linear error equations. In order to establish the error model which adapts to the shipborne environment, the altitude channel needs to be ignored. The state and measurement equation can be established as follows:

(12) $$\{\begin{array}{l} \dot{X}=AX+BW\\ Z=HX+\eta \end{array}$$

where $X={\left[\begin{array}{cccccccccccccc} \phi_{E}^{G} & \phi_{N}^{G} & \phi_{U}^{G} & \delta V_{E}^{G} & \delta V_{N}^{G} & \delta x & \delta y & \delta z & \varepsilon_{bx}^{b} & \varepsilon_{by}^{b} & \varepsilon_{bz}^{b} & \nabla_{bx}^{b} & \nabla_{by}^{b} & \nabla_{bz}^{b} \end{array}\right]}^{T}$, $\phi_{E}^{G}$, $\phi_{N}^{G}$ and $\phi_{U}^{G}$ are the grid attitude error angles; $\delta V_{E}^{G}$, $\delta V_{N}^{G}$ are the grid east and north velocity error; δx, δy, δz are the position errors along the x, y, z axes in e frame; $\varepsilon_{bx}^{b}$, $\varepsilon_{by}^{b}$, $\varepsilon_{bz}^{b}$ are the gyro constant drifts along the x, y, z axes in b frame; $\nabla_{bx}^{b}$, $\nabla_{by}^{b}$, $\nabla_{bz}^{b}$ are the accelerometer constant biases along the x, y, z axes in b frame; $W={\left[\begin{array}{cccccc} \varepsilon_{wx}^{b} & \varepsilon_{wy}^{b} & \varepsilon_{wz}^{b} & \nabla_{wx}^{b} & \nabla_{wy}^{b} & \nabla_{wz}^{b} \end{array}\right]}^{T}$, $\varepsilon_{wx}^{b}$, $\varepsilon_{wy}^{b}$, $\varepsilon_{wz}^{b}$ are the gyro random drifts along the x, y, z axes in b frame; $\nabla_{wx}^{b}$, $\nabla_{wy}^{b}$, $\nabla_{wz}^{b}$ are the accelerometer random biases along the x, y, z axes in b frame.

The difference between the external reference velocity and velocity calculated by the system is taken as the observation. η is the measurement noise array. Coefficient matrix A, system noise driven matrix B, and measurement matrix H are described as follows:

(13) $$A=\left[\begin{array}{ccccc} -(\omega_{iG}^{G}\times ) & {\left(C_{\omega Gv}\right)}_{3,2} & C_{\omega eR}+C_{\omega GR} & -C_{b}^{G} & 0_{3\times 3}\\ {\left(f^{G}\times \right)}_{2,3} & {\left(V^{G}\times C_{\omega Gv}-[(2\omega_{ie}^{G}+\omega_{eG}^{G})\times ]\right)}_{2,2} & {\left(V^{G}\times (C_{\omega GR}+2C_{\omega eR})\right)}_{2,3} & 0_{2\times 3} & {\left(C_{b}^{G}\right)}_{2,3}\\ 0_{3\times 3} & {\left(C_{G}^{e}\right)}_{3,2} & -C_{G}^{e}(V^{G}\times )C_{R} & 0_{3\times 3} & 0_{3\times 3}\\ 0_{3\times 3} & 0_{3\times 2} & 0_{3\times 3} & 0_{3\times 3} & 0_{3\times 3}\\ 0_{3\times 3} & 0_{3\times 2} & 0_{3\times 3} & 0_{3\times 3} & 0_{3\times 3} \end{array}\right]$$

(14) $$B=\left[\begin{array}{c} \begin{array}{cc} -C_{b}^{G} & 0_{3\times 3}\\ 0_{2\times 3} & {\left(C_{b}^{G}\right)}_{2,3} \end{array}\\ 0_{9\times 6} \end{array}\right]$$

(15) $$H=\left[\begin{array}{ccc} 0_{2\times 3} & \begin{array}{cc} 1 & 0\\ 0 & 1 \end{array} & 0_{2\times 9} \end{array}\right]$$

where the Vector× means the skew-symmetric matrix related to the Vector, the ${\left(matrix\right)}_{m,n}$ means the first m rows and first n columns of the matrix. The detailed expressions of submatrices $C_{\omega Gv}$, $C_{\omega eR}$, $C_{\omega GR}$ and $C_{R}$ can refer to the reference [16].

### 3.2. Kalman Filtering and Feedback Correction

Kalman filtering is a technology based on the minimum variance criteria, and the states are estimated according to the state and measurement equation. The equations of the discrete Kalman filtering are described as follows, and the detailed definition of each symbol can be seen in reference [29].

(16) $$\begin{array}{c} {\hat{X}}_{k/k-1}=\phi_{k,k-1}{\hat{X}}_{k-1}\\ P_{k/k-1}=\phi_{k,k-1}P_{k-1}\phi_{k,k-1}^{T}+\Gamma_{k-1}Q_{k-1}\Gamma_{k-1}^{T}\\ K_{k}=P_{k/k-1}H_{k}^{T}{\left(H_{k}P_{k/k-1}H_{k}^{T}+R_{k}\right)}^{-1}\\ {\hat{X}}_{k}={\hat{X}}_{k/k-1}+K_{k}(Z_{k}-H_{k}{\hat{X}}_{k/k-1})\\ P_{k}=(I-K_{k}H_{k})P_{k/k-1} \end{array}$$

$\phi_{k,k-1}$, $\Gamma_{k-1}$, $H_{k}$, and $Z_{k}$ are determined after the discretizing the Equation (12), when the initializations of the state vector X and P matrix are finished, the Kalman filtering can be conducted. Section 3.1 has given the error model of the shipborne grid SINS, and the error model of the DVL should be considered as well. In practice, the velocity measured by DVL can easily be affected by the external environment. On one hand, the velocity measured by DVL is easily polluted by the ocean currents when the ship is maneuvering; on the other hand, in some sea areas, such as the gulfs and trenches, the DVL measurement error is sensitive to the changes of the ocean currents. For the reasons above, establishing a precise error model of the DVL becomes difficult. In order to solve this problem, when the external reference velocity is available, combining with the actual situation, the error model of the external velocity can be simplified as a constant in a certain period of time. In this way, when the external velocity is available, supposing that the external velocity error is a constant, the grid SINS works in an external level damping state; otherwise the grid SINS works in a non-damping state. Therefore, the key to the question is to find out an effective criterion to judge if the velocity measured by DVL is available.

The criterion can be established according to the changes of the observation. The observation can be described as: $Z=V_{GSINS}-V_{DVL}$, $V_{GSINS}$ represents the velocity calculated by grid SINS, and $V_{DVL}$ represents the velocity measured by DVL. $V_{GSINS}$ and $V_{DVL}$ can be described as follows: $V_{GSINS}=V+\delta V_{GSINS}$ and $V_{DVL}=V+\delta V_{DVL}$, where V is the true velocity of the ship, $\delta V_{GSINS}$ represents the velocity error of the grid SINS, and $\delta V_{DVL}$ represents the velocity measurement error of the DVL. The observation Z can be described as:

(17) $$Z=V_{GSINS}-V_{DVL}=\delta V_{GSINS}-\delta V_{DVL}$$

$Z_{1}$ and $Z_{2}$ represent the observation at time $t_{1}$ and $t_{2}$, respectively. The difference between $Z_{1}$ and $Z_{2}$ can be obtained as:

(18) $$\delta Z=Z_{1}-Z_{2}=(\delta V_{GSINS}^{1}-\delta V_{GSINS}^{2})-(\delta V_{DVL}^{1}-\delta V_{DVL}^{2})$$

where $\delta V_{GSINS}^{1}-\delta V_{GSINS}^{2}$ is the difference between the velocity errors of grid SINS at time $t_{1}$ and $t_{2}$; $\delta V_{DVL}^{1}-\delta V_{DVL}^{2}$ is the difference between the velocity measurement errors of DVL at time $t_{1}$ and $t_{2}$. Obviously, $\delta V_{GSINS}^{1}-\delta V_{GSINS}^{2}$ will not change dramatically in a short period of time, thus, as long as δZ changes dramatically, which means the external velocity measurement error has greatly changed as a result of the ship’s maneuvering or changes of the sea conditions, in this case, the external velocity measured by DVL cannot be used. On the basis of above-mentioned analysis, when δZ changes dramatically in a certain period of time, the grid SINS works in the non-damping state, otherwise the grid SINS works in the external level damping state.

When DVL can provide an accurate reference velocity, the grid level velocity errors, position errors and grid level attitude error angles can all be accurately estimated. The attitude, velocity, and position errors of grid SINS can be compensated by using these three kinds of estimates to calibrate the system. However, as a result of the measurement error of the DVL, the observability of the state variables changes. Practical tests show that grid level velocity errors and position errors cannot be estimated accurately when the reference velocity contains constant error, but the grid level attitude error angles can still be accurately estimated. In this way, only the estimated grid level attitude error angles can be used to calibrate the system.

**Figure 3:**
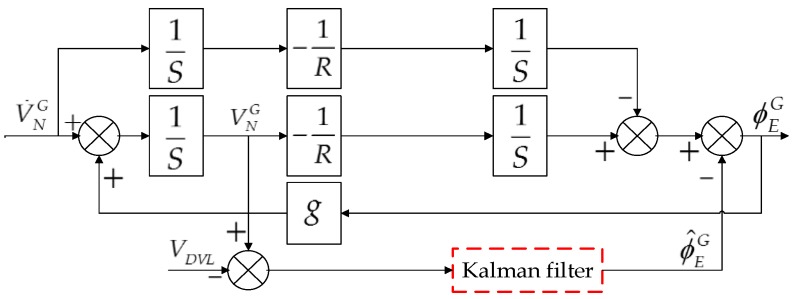
Block diagram of KF-DGSINS algorithm.

Two correction methods are usually used to calibrate the system: one is feedback correction and the other is output correction. Obviously, if output correction is selected, only the oscillation errors of the grid level attitude angles can be suppressed, as a result of the system’s open-loop structure. Feedback correction is the suitable way to calibrate the system for this method, and can suppress not only the oscillation errors of the grid level attitude angles, but also the oscillation errors of the grid level velocity and position. In fact, the existence of the level attitude error angles is the essential reason for the Schuler periodic oscillation, so the Schuler oscillation errors can be suppressed effectively through the feedback correction. Here we take the grid north level circuit as an example and the block diagram of the KF-DGSINS algorithm is shown in Figure 3. In Figure 3, S represents the variable after the Laplace transformation, and $\frac{1}{S}$ means the integration process. R represents the radius of the earth. ${\hat{\phi }}_{E}^{G}$ represents the estimate of the grid east attitude error angle.

## 4. The T-DGSINS Algorithm

**Figure 4:**
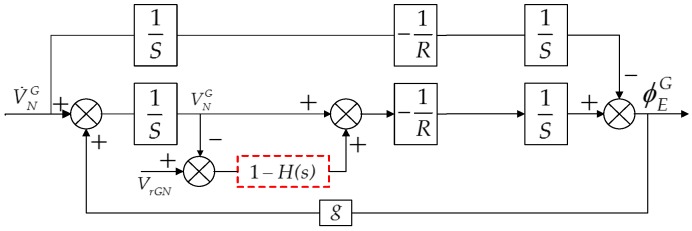
Block diagram of T-DGSINS algorithm.

Referring to the traditional external level damping algorithm based on the damping network of common SINS, the T-DGSINS based on classical control theory can be designed. Taking the grid north level circuit as an example, the block diagram of the T-DGSINS is shown in Figure 4. The external reference velocity is introduced to the system through the damping network 1−H(s), from the physical sense, the introduction of the external reference velocity just adds a compensation channel to the system, and the navigation accuracy of this method is sensitive to the error of the external reference velocity.

The T-DGSINS based on the damping network can effectively dampen the Schuler oscillation errors of the grid SINS, but several obvious disadvantages of this method are difficult to overcome:

1. The damping network added to the system breaks the applicability of the Schuler adjustment condition, and an equivalent step-function signal is introduced to the system when the state of the grid SINS is switched from the non-damping state to the external level damping state. Therefore, the error overshoot phenomenon exists in the state switching process, and the navigation results cannot be used for a certain period of time.
2. T-DGSINS is designed based on the conditions of a single-input-single-output system (SISO), so the coupling effects of each calculating circuit cannot be solved.

## 5. Simulation Results and Discussions

This section is divided into two parts: one is the simulation results’ analysis, the other is the semi-physical simulation results’ analysis. The simulation results demonstrated the theoretical feasibility of the KF-DGSINS, and the semi-physical simulation results confirmed the validity of the KF-DGSINS in practical application.

### 5.1. Simulation Results and Discussions

**Figure 5:**
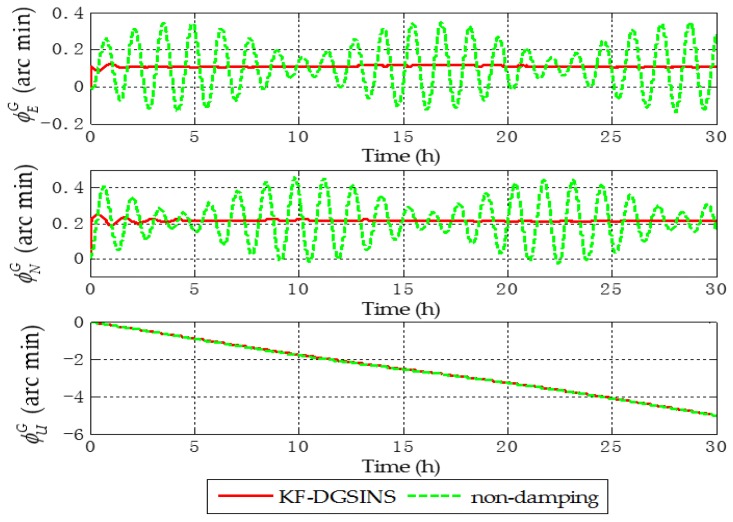
Grid attitude angle errors of the simulation results.

**Figure 6:**
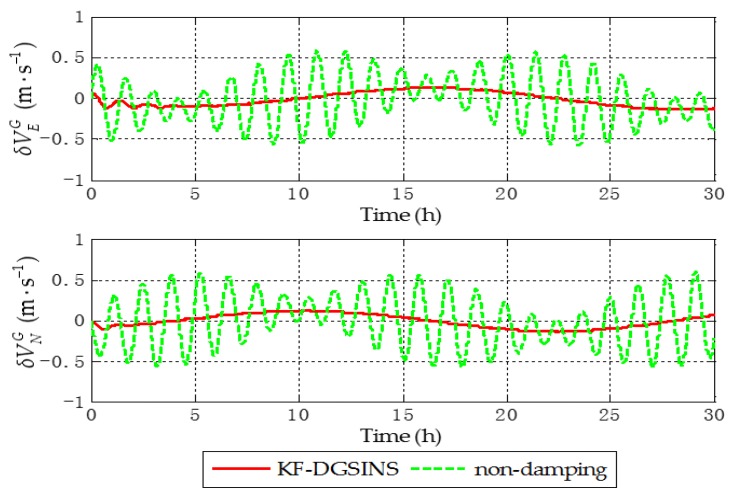
Grid level velocity errors of the simulation results.

**Figure 7:**
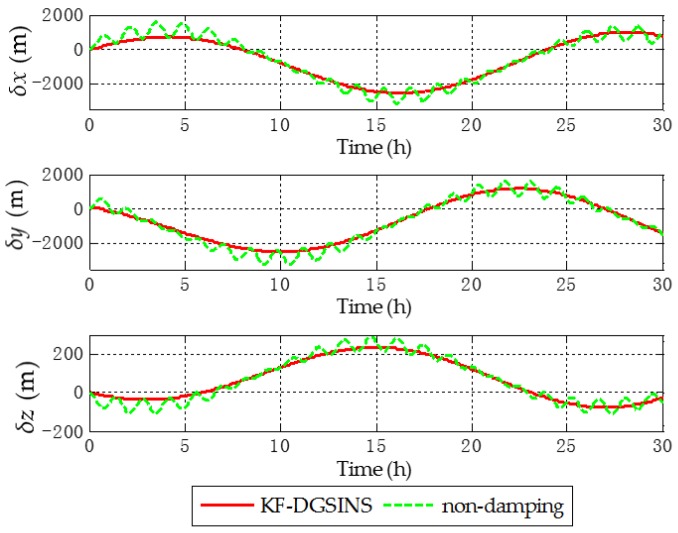
Position errors in e frame of the simulation results.

Aiming at the grid SINS, in order to test the damping performance of the KF-DGSINS, non-damping grid SINS and KF-DGSINS are simulated on a stationary base. Gyroscopic and accelerometer outputs are generated by the track generator. The general idea of the track generator is: according to the change rules of the carrier’s position, velocity, attitude, and the settings of the IMU error, the outputs of the gyroscope and accelerometer can be back-stepped through the SINS mechanization. The simulation conditions are set as follows: simulation lasts 30 h; the initial latitude and longitude are 85° N and 18° E; the three-axis gyro constant drifts are set as 0.003 degree per hour and the three-axis accelerometer constant biases are set as 0.00005 g, and the random error of the gyro and accelerometer are set as white noise; the error of the external reference velocity is set as white noise. The simulation results are shown in Figure 5, Figure 6 and Figure 7.

The dotted green line is the error curve of the non-damping grid SINS; the solid red line is the error curve of the grid SINS, adopting the KF-DGSINS. The simulation results show that the Schuler periodic oscillation exists in the errors of grid level attitude, grid level velocity, and position. The proposed KF-DGSINS effectively suppressed the Schuler periodic oscillation, and at the same time the Foucault periodic oscillation, which modulates the Schuler periodic oscillation. For grid level attitude errors, only the constant errors caused by accelerometer biases remain; for grid level velocity errors and position errors, the 24 h earth periodic oscillation still exists in the system. The error of the grid course angle is mainly influenced by the earth periodic oscillation, therefore, non-damping grid SINS and KF-DGSINS achieved almost the same effect. In conclusion, aiming at ships which have the external reference information to conduct a system reset regularly, the KF-DGSINS can observably improve the navigation accuracy of the grid SINS.

When the state of grid SINS is switched from the non-damping state to the external level damping state, big overshoot errors are introduced to the system by using the T-DGSINS. The KF-DGSINS proposed in this paper can restrain the overshoot errors effectively. In order to verify the advantage of the KF-DGSINS, the T-DGSINS and the KF-DGSINS are simulated under the same conditions. The simulation conditions are set as follows: gyro and accelerometer outputs are generated by the track generator; simulation lasts 10 h; the initial latitude and longitude are 85° N and 18° E; the ship sails eastward along the latitude circle and the velocity is 10 m/s; the swing of the ship is set as the sine function:

(19) $$\{\begin{array}{l} pitch=pitch_{m}\sin(2\pi /T_{P}+Ph_{P})\\ roll=roll_{m}\sin(2\pi /T_{R}+Ph_{R})\\ heading=90{}^{\circ}+heading_{m}\sin(2\pi /T_{H}+Ph_{H}) \end{array}$$

where $pitch_{m}$, $roll_{m}$, $heading_{m}$ are swing amplitudes, and $pitch_{m}=3{}^{\circ}$, $roll_{m}=5{}^{\circ}$, $heading_{m}=4{}^{\circ}$; $T_{P}$, $T_{R}$, $T_{H}$ are swing periods, and $T_{P}=7\;s$, $T_{R}=9\;s$, $T_{H}=12\;s$; $Ph_{P}$, $Ph_{R}$, $Ph_{H}$ are initial phases and they are random values in the range from $0^{{}^{\circ}}$ to $90^{{}^{\circ}}$. 

**Figure 8:**
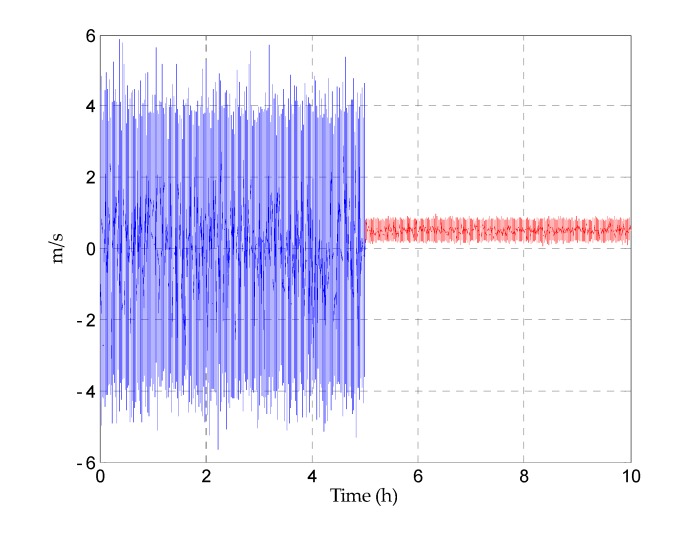
The error of the grid east reference velocity.

**Figure 9:**
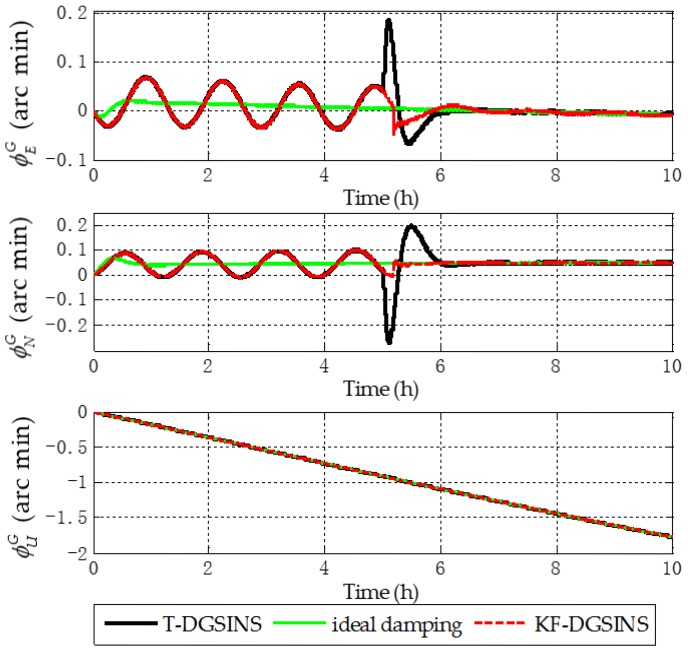
Grid attitude angle errors of the simulation results.

**Figure 10:**
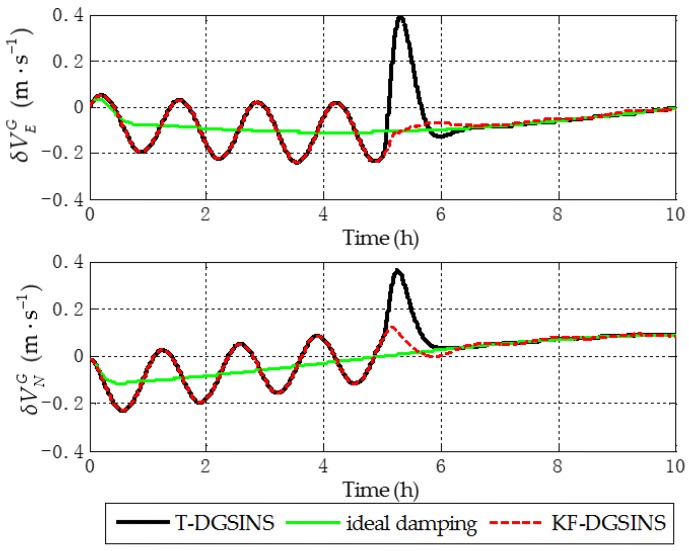
Grid level velocity errors of the simulation results.

**Figure 11:**
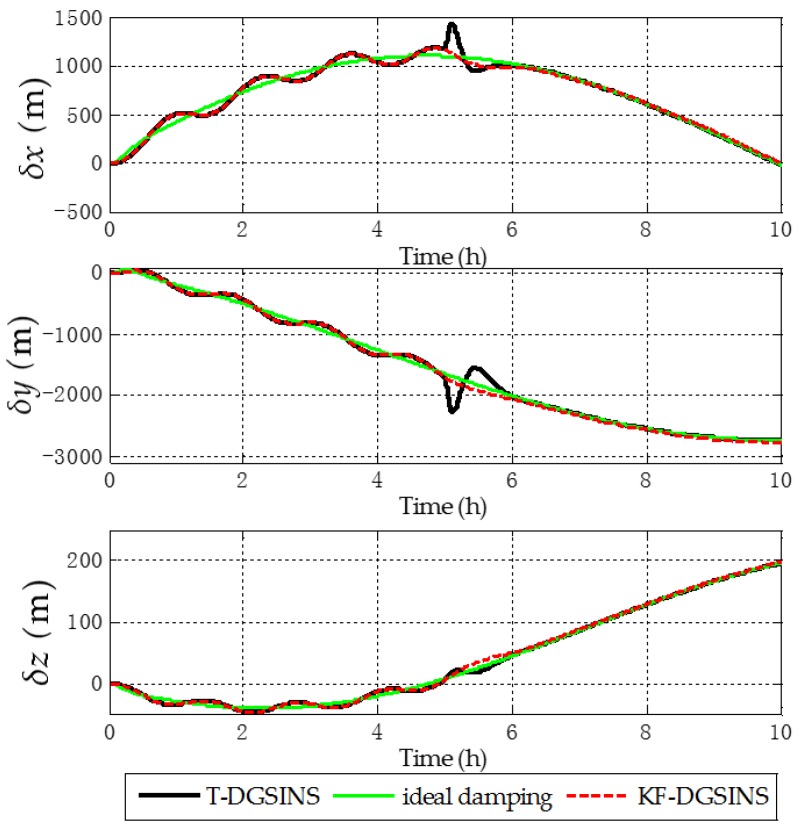
Position errors in e frame of the simulation results.

In practice, the velocity measured by DVL is unavailable when the ship maneuvers or sails in some special sea areas. When the ship sails in a steady state, according to the previous introduction, the error of the DVL can be simplified as a constant, and the velocity measured by DVL is available. Considering the practical situation, the errors of the grid east and north velocity can be set as follows: before t=5 h, the velocity measured by DVL is set to be unavailable. In order to simulate the condition that the error of the external reference velocity changes dramatically, the errors are set as the zero mean white noise with large amplitudes; after t=5 h, the velocity measured by DVL is set to be available, and the error model of the velocity measured by DVL is simplified as the constant. In this case, the errors are set as the sum of the white noise and the constant (1kn). Taking the error of the grid east reference velocity as an example, the error of the grid east reference velocity is shown in Figure 8. When the reference velocity error is set as zero, the results of the T-DGSINS represent the ideal damping results of the grid SINS. In this way, in order to better reflect the advantage of the proposed KF-DGSINS, the ideal damping results are given when the reference velocity error is set as zero from beginning to end. The simulation results are shown in Figure 9, Figure 10 and Figure 11.

The solid green line is the ideal damping error curve of the grid SINS; the solid black line is the error curve of the grid SINS, adopting the T-DGSINS; the dotted red line is the error curve of the grid SINS, adopting the KF-DGSINS. Before t=5 h, the error of the reference velocity changes dramatically. According to the criterion which is introduced in Section 3.2, the external reference velocity cannot be used. Therefore, the grid SINS works in the non-damping state; after t=5 h, the external reference velocity changes slightly. According to the criterion, the external reference velocity becomes available, therefore, the grid SINS works in an external level damping state. What needs to be stressed is that the feedback correction should start after the process of Kalman filtering becomes stable. Some conclusions can be made from the simulation results: (1) two kinds of algorithm can effectively suppress the Schuler oscillation errors, and in the state switching process, the overshoot errors of the KF-DGSINS are obviously smaller than the overshoot errors of the T-DGSINS. For T-DGSINS, when the state of system is switched from the non-damping state to the external level damping state, the damping network is added to the level circuit of the grid SINS, and an equivalent step-function signal is introduced to the system. In this case, the equilibrium state of the system is broken, and the overshoot phenomenon occurs. For KF-DGSINS, no damping network is added to the system. When the Kalman filter is stable, the feedback correction of the grid level attitude error angles can suppress the Schuler oscillation error without breaking the equilibrium state of the system, and this is the reason why KF-DGSINS has a better performance in restraining the overshoot phenomenon than T-DGSINS; (2) the grid course angle error is hardly influenced by the overshoot phenomenon, therefore, T-DGSINS and KF-DGSINS achieved almost the same effect; (3) when the damping state is stable, the damping results of the KF-DGSINS and T-DGSINS are almost the same as the ideal damping results, which means the constant error of the external reference velocity will not affect the damping results of the KF-DGSINS and T-DGSINS when the damping state is stable. In a word, compared with the T-DGSINS, the KF-DGSINS has a better performance in overcoming the overshoot errors and this algorithm effectively improves the navigation accuracy of the grid SINS in the state switching process.

### 5.2. Semi-Physical Simulation Results and Discussions

Due to the limit of the geographic restriction, the semi-physical simulation is conducted to verify the performance of the proposed algorithm in practice. The outputs of the gyroscopes and accelerometers can be described as follows:

(20) $$\{\begin{array}{l} \omega_{ib\_real}^{b}=\omega_{ib\_ideal}^{b}+\omega_{ib\_error}^{b}\\ f_{\_real}^{b}=f_{\_ideal}^{b}+f_{\_error}^{b} \end{array}$$

where $\omega_{ib\_real}^{b}$ and $f_{\_real}^{b}$ are the measurements of the gyroscopes and accelerometers, respectively; $\omega_{ib\_ideal}^{b}$ and $f_{\_ideal}^{b}$ are the ideal measurements of the gyroscopes and accelerometers, respectively; $\omega_{ib\_error}^{b}$ are the measurement errors of the gyroscopes, and $f_{\_error}^{b}$ are the measurement errors of the accelerometers.

**Figure 12:**
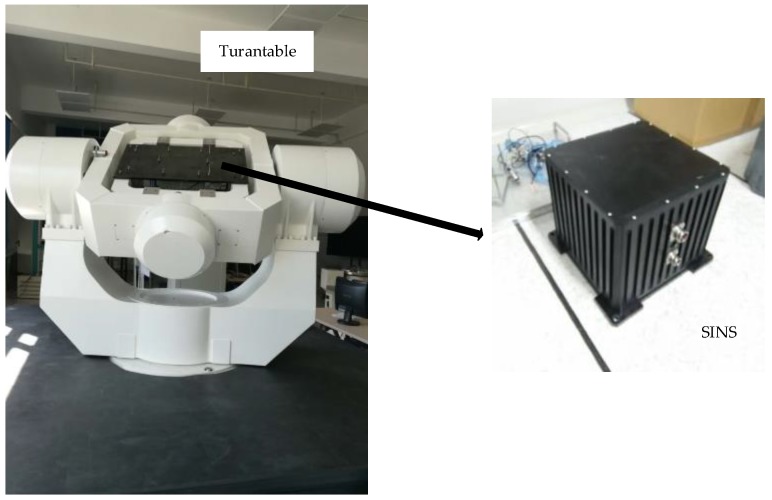
Static IMU data collection system.

In order to get more realistic simulation measurements of the gyroscopes and the accelerometers in polar regions, the key point is to get the measurement errors of the gyroscopes and the accelerometers. Once the initial position, velocity, attitude, and the form of motion are determined, $\omega_{ib\_ideal}^{b}$ and $f_{\_ideal}^{b}$ can be generated by the track generator. With the aid of the high-precision three-axis turntable, $\omega_{ib\_error}^{b}$ and $f_{\_error}^{b}$ can be obtained easily. Collecting the IMU data was done in static state, and the static IMU data collection system is shown in Figure 12.

The SINS is installed on the three-axis turntable, and the attitude and position of the turntable are precisely known. The experimental data is provided by the IMU. The high-precision three-axis turntable can provide the accurate attitude reference of the carrier. With the aid of the high-precision three-axis turntable, the carrier can move in preset attitude change rules. The main technical indexes of the SGT-8 three-axis turntable in this experiment are shown in Table 1.

In static state, the three-axis gyroscopes are only sensitive to the rotational angular velocity of the earth, and the three-axis accelerometers are only sensitive to the local acceleration of gravity. Therefore, $\omega_{ib\_error}^{b}$ and $f_{\_error}^{b}$ can be calculated by using (21):

(21) $$\{\begin{array}{l} \omega_{ib\_error}^{b}=\omega_{ie\_real}^{b}-\omega_{ie}^{b}\\ f_{\_error}^{b}=f_{\_real}^{b}-f^{b} \end{array}$$

where $\omega_{ie\_real}^{b}$ and $f_{\_real}^{b}$ are the measurements of the gyroscopes and accelerometers in static state; $\omega_{ie}^{b}$ and $f^{b}$ can be calculated by using the precise position and attitude of the turntable.

The related parameters of the gyroscopes and accelerometers can be roughly calculated from the static test, and they are shown as follows: the three-axis gyro constant drifts are $2.573\times 10^{-8}\;\mathrm{rad}/s$, $-2.607\times 10^{-8}\;\mathrm{rad}/s$, $2.387\times 10^{-8}\;\mathrm{rad}/s$, respectively; the three-axis gyro random drift variances are ${\left(2.9938\times 10^{-6}\;\mathrm{rad}/s\right)}^{2}$, ${\left(2.4866\times 10^{-6}\;\mathrm{rad}/s\right)}^{2}$, ${\left(3.2143\times 10^{-6}\;\mathrm{rad}/s\right)}^{2}$, respectively; the three-axis accelerometer constant biases are $3.214\times 10^{-4}\;{m/s}^{2}$, $2.847\times 10^{-4}\;{m/s}^{2}$, $-3.072\times 10^{-4}\;{m/s}^{2}$, respectively; the three-axis accelerometer random biases variances are ${\left(0.001594\;{m/s}^{2}\right)}^{2}$, ${\left(0.001771\;{m/s}^{2}\right)}^{2}$, ${\left(0.001817\;{m/s}^{2}\right)}^{2}$, respectively.

**Figure 13:**
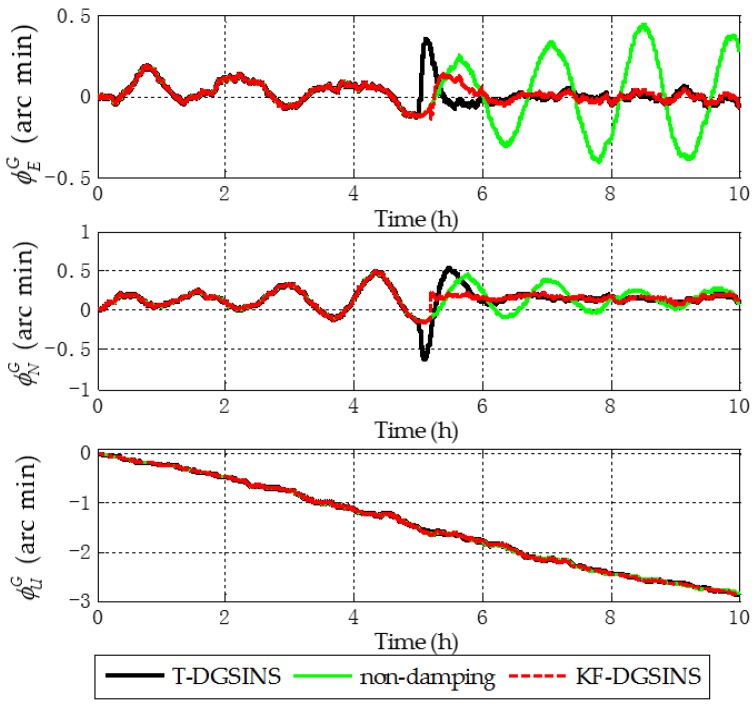
Grid attitude angle errors of the semi-physical simulation results.

**Figure 14:**
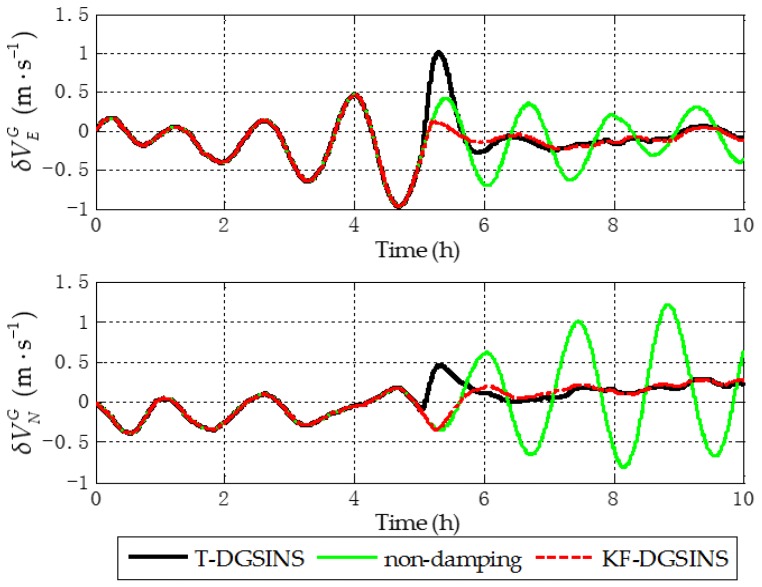
Grid level velocity errors of the semi-physical simulation results.

**Figure 15:**
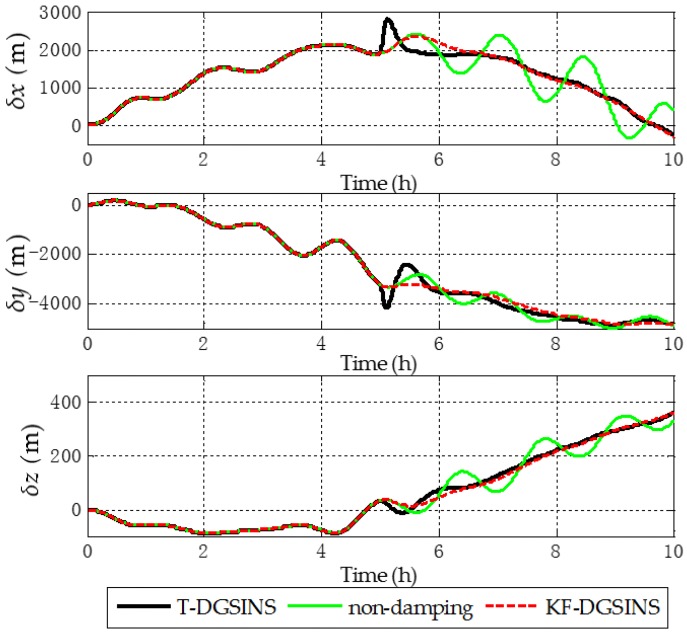
Position errors in e frame of the semi-physical simulation results.

The simulation conditions are set as follows: simulation lasts 10 h; the initial latitude and longitude are 85° N and 18° E; the ship sails eastward along the latitude circle and the velocity is 10 m/s; the swing of the ship is set as (18), and each parameter is the same as before. Due to the changes of the external velocity errors, before t=5 h, the external velocity is set to be unavailable, and the grid SINS works in the non-damping state; after t=5 h, the external velocity is available with constant errors, and the system works in the external level damping state. The results of the semi-physical simulation are shown in Figure 13, Figure 14 and Figure 15.

The solid green line is the error curve of the non-damping grid SINS; the solid black line is the error curve of the grid SINS, adopting the T-DGSINS; the dotted red line is the error curve of the grid SINS, adopting the KF-DGSINS. As shown in Figure 13, Figure 14 and Figure 15, the semi-physical simulation results further verify that the proposed KF-DGSINS has a better performance than the T-DGSINS. The results of the semi-physical simulation coincide with the results of the simulation results: on one hand, the KF-DGSINS and the T-DGSINS can suppress the Schuler oscillation errors effectively, on the other hand, in the state switching process, for KF-DGSINS, as a result of no damping network being added to the circuit, the equilibrium state of the grid SINS maintains, so the overshoot errors of the KF-DGSINS are obviously smaller than the overshoot errors of the T-DGSINS, and this effectively improves the navigation accuracy of the grid SINS in the state switching process. Grid course angle error is hardly influenced by the overshoot phenomenon, therefore T-DGSINS and KF-DGSINS achieved almost the same effect. The KF-DGSINS has a better performance in overcoming the overshoot errors when the state of grid SINS is switched from the non-damping state to the external level damping state, therefore, the shipborne grid SINS based on the KF-DGSINS is feasible for ships working in polar regions.

## 6. Conclusions

Aiming at ships which have the external reference information to conduct a system reset regularly, non-damping and external level damping working states are two kinds of frequently-used working states for ships sailing in polar regions. Therefore, the working state of the ship is frequently switched between the two working states mentioned above. In order to overcome the overshoot phenomenon of the T-DGSINS in the state switching process, the KF-DGSINS is proposed in this paper. The simulation results show that the KF-DGSINS can achieve the effective suppression of the Schuler oscillation errors, and can also reduce the overshoot errors in the state switching process. In conclusion, in view of the advantages of the KF-DGSINS, the KF-DGSINS can satisfy the needs of ships sailing in polar regions.

## Figures and Tables

**Table 1:** The technical indexes of the SGT-8 three-axis turntable.

	Outer Axis	Middle Axis	Inner Axis	Unit
Location accuracy of angular position	±3	±2	±2	arc sec
Maximum angular rate	±180	±250	±400	°/s
Rotation rate resolution	0.001	0.001	0.001	°/s
